# Genetic diversity analysis in the Brazilian Amazon reveals a new evolutionary lineage and new karyotype for the genus *Mesomys* (Rodentia, Echimyidae, Eumysopinae)

**DOI:** 10.1371/journal.pone.0291797

**Published:** 2023-10-04

**Authors:** Leony Dias de Oliveira, Willam Oliveira da Silva, Marlyson Jeremias Rodrigues da Costa, Jeferson Costa Carneiro, Iracilda Sampaio, Juliane Saldanha da Silva, Rogério Vieira Rossi, Ana Cristina Mendes-Oliveira, Julio Cesar Pieczarka, Cleusa Yoshiko Nagamachi

**Affiliations:** 1 Centro de Estudos Avançados da Biodiversidade, Laboratório de Citogenética, ICB, Universidade Federal do Pará, Belém, Pará, Brazil; 2 Genômica e Biologia de Sistemas, Universidade Federal do Pará, Belém, Pará, Brazil; 3 Laboratório de Mastozoologia, Instituto de Biociências, Universidade Federal do Mato Grosso, Cuiabá, Brazil; 4 Laboratório de Zoologia e Ecologia de Vertebrados, ICB, Universidade Federal do Pará, Belém, Pará, Brazil; Instituto Leonidas e Maria Deane Fiocruz Amazonia, BRAZIL

## Abstract

Morphological, molecular and chromosomal studies in the genera *Lonchothrix* and *Mesomys* have contributed to a better understanding of taxonomic design, phylogenetic relationships and karyotypic patterns. Recent molecular investigations have shown a yet undescribed diversity, suggesting that these taxa are even more diverse than previously assumed. Furthermore, some authors have questioned the limits of geographic distribution in the Amazon region for the species *M*. *hispidus* and *M*. *stimulax*. In this sense, the current study sought to understand the karyotypic evolution and geographic limits of the genus *Mesomys*, based on classical (G- and C-banding) and molecular cytogenetic analysis (FISH using rDNA 18S and telomeric probes) and through the sequencing of mitochondrial genes Cytochrome b (Cytb) and Cytochrome Oxidase—Subunit I (CO using phylogeny, species delimitation and time of divergence, from samples of different locations in the Brazilian Amazon. The species *M*. *stimulax* and *Mesomys* sp. presented 2n = 60/FN = 110, while *M*. *hispidus* presented 2n = 60/FN = 112, hitherto unpublished. Molecular dating showed that *Mesomys* diversification occurred during the Plio-Pleistocene period, with *M*. *occultus* diverging at around 5.1 Ma, followed by *Mesomys* sp. (4.1 Ma) and, more recently, the separation between *M*. *hispidus* and *M*. *stimulax* (3.5 Ma). The ABGD and ASAP species delimiters support the formation of 7 and 8 potential species of the genus *Mesomys*, respectively. Furthermore, in both analyzes *Mesomys* sp. was recovered as a valid species. Our multidisciplinary approach involving karyotypic, molecular and biogeographic analysis is the first performed in *Mesomys*, with the description of a new karyotype for *M*. *hispidus*, a new independent lineage for the genus and new distribution data for *M*. *hispidus* and *M*. *stimulax*.

## Introduction

Considered the largest tropical rainforest, the Amazon is home to a diversity of ecosystems that allow a high abundance and richness of species. In this sense, several studies have sought to understand the distribution and processes that gave rise to the diversity of species and the biogeographic patterns observed in the region. From this, biogeographic units were recognized for the Amazon, whose main characteristic is an evolutionary history of the species that occur in these areas [1–3]. Eight areas of endemism for terrestrial vertebrates are currently recognized in the Amazon biome, namely: Napo, Imeri, Guiana, Inambari, Rondônia, Tapajós, Xingu and Belém [4]. These regions are delimited by the great Amazonian rivers and characterized as units that reflect the evolutionary history of the species found there [4, 5].

Among the groups of terrestrial vertebrates, the rodents of the family Echimyidae are considered the most diverse South American hystricognaths, comprising 95 species grouped in 21 genera. This family is widely distributed in the Amazon rainforest, occurring in all recognized areas of endemism [6]. Investigations with mitochondrial genes Cytochrome b (Cytb) and Cytochrome C Oxidase—Subunidade I (CO1) and nuclear genes Apolipoprotein B (apoB), Interphotoreceptor Retinoid-Binding Protein (IRBP), Recombination Activating 1 (RAG1) and Von Willebrand Factor (vWF) fragments carried out in Echimyidae have resulted in the recognition of three or four subfamilies, depending on the author and analyzes performed [6–10].

The subfamily Eumysopinae is composed of nine genera, four of which are terrestrial (*Hoplomys*, *Proechimys*, *Thrichomys* and *Trinomys*), three are semifossorial (*Carterodon*, *Clyomys* and *Euryzygomatomys*), and two are arboreal (*Mesomys* and *Lonchothrix*) [6]. The genus *Mesomys* comprises four species (*M*. *hispidus*, *M*. *stimulax*, *M*. *occultus* and *M*. *leniceps*), which are widely distributed in the Brazilian Amazon and regions from the Guianas to the eastern part of the Andes. *Mesomys hispidus* occurs widely in the Amazon region, except on the right bank of the lower Tapajós river south of the Amazonas river [6]. *Mesomys stimulax* occurs from the eastern Amazon south of the river Amazonas, particularly on the right bank of the lower Tapajós river up to the right bank of the Tocantins river and may be sympatric with *M*. *hispidus* on the right bank of the upper Tapajós river [6]. However, there are inconsistencies regarding the limits of distribution and sympatry between *M*. *hispidus* and *M*. *stimulax*, given that sympatry zones were described for these species on the left bank of the lower Tapajós river south of the Amazon river [11].

In view of the difficulties in taxonomic identification, mainly associated with the overlapping of ecological and morphological characteristics and regions of sympatry, studies have sought to employ different methods for a better delineation of taxa, associating morphological [9, 10], molecular [6–10], and karyotypic data [11, 12]. Analysis of Cytb sequences and morphometric data from *M*. *hispidus* samples demonstrated a high intraspecific divergence with the structuring of different clades, associated with variation in cranio-dental size, suggesting that it is a species complex [10]. Despite this variation, the authors allocated all forms provisionally detected within *M*. *hispidus*, but suggested that studies using morphological, molecular and chromosomal analyzes should be carried out.

The karyotypic date described for the genus *Mesomys* reveal the presence of a karyotype for *M*. *occultus* with a diploid number (2n) of 42 chromosomes and a fundamental number (FNa) of 54 [9]. Similarly, a distinct karyotype was identified for *M*. *hispidus* with 2n = 60 and FNa = 116 [9, 11]. Additionally, two cytotypes have been recorded for *M*. *stimulax* exhibiting 2n = 60 and FNa = 110 or 116 [9, 12]. Recently, chromosomal G- and C-banding and fluorescent *in situ* hybridization (FISH) analyses using telomeric and rDNA probes were performed on samples of *M*. *hispidus* (2n = 60/ FNa = 116 from the Xingu endemic region [11], as well as on *M*. *stimulax* (2n = 60/FNa = 110) collected in the Tapajós endemic area [12] (S1 Table).

Considering the underestimated genetic diversity and inconsistencies in geographic limits in the genus *Mesomys*, the present study analyzed samples of *Mesomys* from different locations in the Brazilian Amazon by classical and molecular cytogenetics, through G- and C- banding and FISH with telomeric and 18S rDNA probes and the phylogenetic relationship of the genus using Cytb and COI genes fragments, in addition to species delimiters and timetree. We describe possible chromosomal rearrangements that contributed to evolution and diversification, in addition to investigating the distribution of the species *M*. *hispidus* and *M*. *stimulax* from our samples distributed in the Brazilian Amazon, correlating with areas of endemism.

## Materials and methods

### Ethics statement

The specimens were kept stress-free with full access to food and water until euthanasia was performed in accordance with animal welfare guidelines established by Brazilian resolution CFMV n.1000/2012 and CFBio n. 301/2012. The necessary euthanasia was performed by intraperitoneal injection of buffered and diluted barbiturates after local anesthesia, in accordance with animal welfare guidelines established by the Animal Ethics Committee (Comitê de Ética Animal) from Universidade Federal do Pará (UFPA), which authorized the present study (Permit 68–2015). JCP has a permanent scientific collecting permit (number 13248) from “Instituto Chico Mendes de Conservação da Biodiversidade”. The Cytogenetics Laboratory from UFPA has a special permit to sample transport (number 19/2003) and to use the samples for research purposes (number 52/2003) from the Ministry of Environment.

### Samples

**Fig 1:**
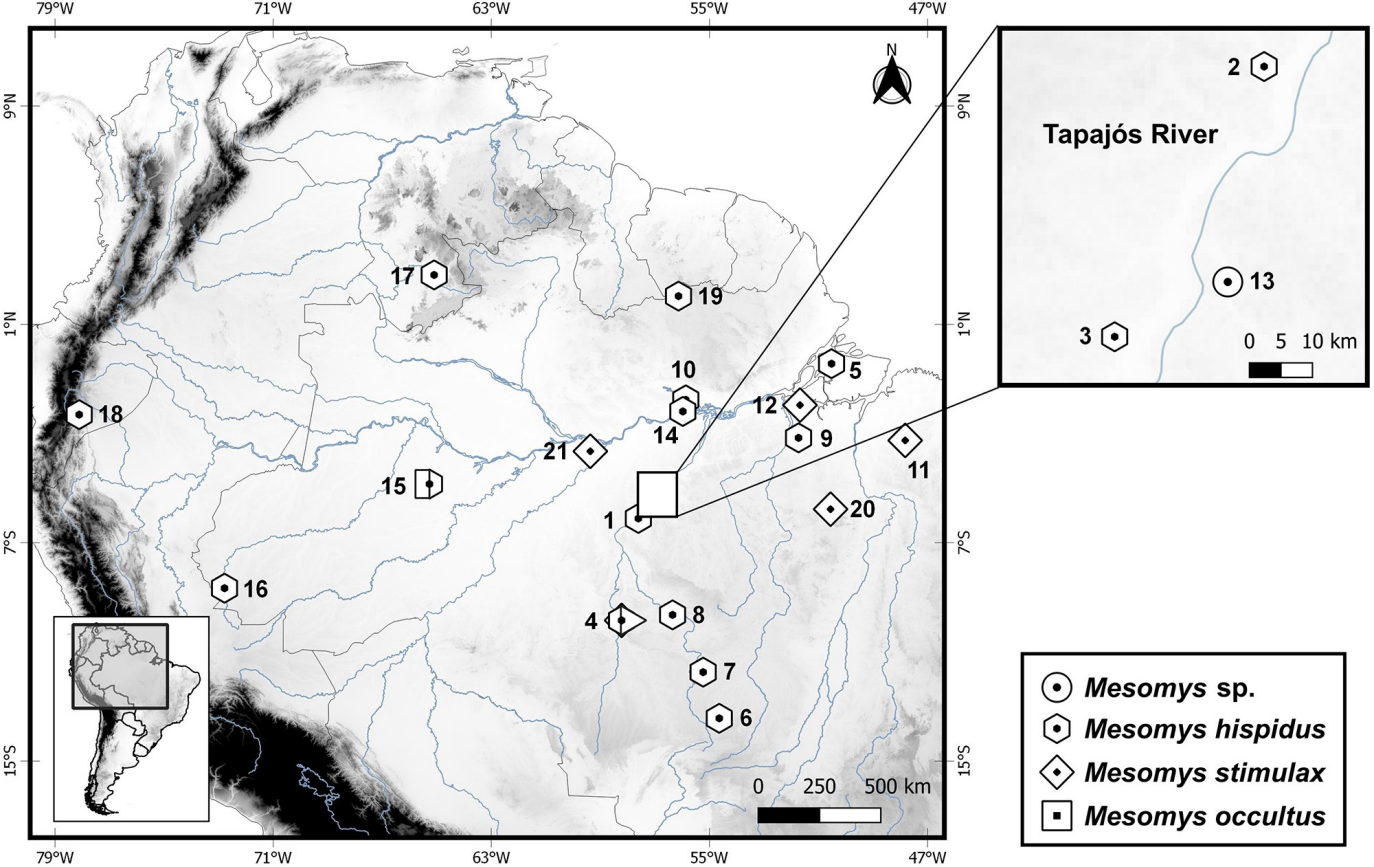
Map with the collection points of the samples of the *Mesomys* genus whose sequences or karyotypes were employed in this study. Present study: *M*. *hispidus* (1 to 10), *M*. *stimulax* (4, 11 and 12) and *Mesomys* sp. (13). Literature and GenBank: *M*. *hispidus* (14 to 19), *M*. *stimulax* (20 and 21), *M*. *occultus* (15). The numbers refer to the locations of each sample. More detailed information can be found in S1 and S2 Tables. Circle: *Mesomys* sp.; hexagon: *M*. *hispidus*; diamond: *M*. *stimulax*; and square: *M*. *occultus*. This map was made using the free software Quantum-Gis version 2.10.1. The databases were obtained from DIVA-GIS. The files provided by DIVA-GIS are free of charge.

We obtained karyotypes from a female specimen of *M*. *hispidus* from Jacareacanga (locality 1, Fig 1); a male specimen of *M*. *stimulax* from Paragominas (locality 11, Fig 1); and a male specimen of *Mesomys* sp. from Itaituba (locality 13, Fig 1). Specimens were captured using pitfall traps [13]. All localities are in the state of Pará, Brazil (Fig 1; S1 Table).

The molecular analysis of the present study used 16 tissue samples from *Mesomys* for DNA extraction to obtain sequences of the mitochondrial genes (Cytb and COI). The data were complemented with sequences available in GenBank (Fig 1; S2 Table).

### Molecular analysis

Total DNA extraction was performed using Promega’s Wizard Genomic Kit, according to the manufacturer’s protocol. PCRs were standardized with 15 μl of final volume, containing ~30ng of genomic DNA; 2.4 μl of dNTPs (1.25mM); 1.5 μl 10X buffer (200 mM Tris-HCl, 500 mM KCl); 1 μl MgCl2 (25 mM); 1 μl of each primer (0.2 μM) and 1 U of Taq DNA polymerase. With the exception of the annealing temperature of the primers, the other steps of the amplification protocol were identical for the two markers, with the following conditions: initial denaturation at 95°C for 5 min, with 35 cycles of denaturation at 94°C for 30 s, hybridization temperature for 40 s, extension of 72° C for 40 s, and final extension at 72°C for 5 min. Amplifications of 792 bp of the mitochondrial Cytochrome b gene were performed with primers 5’-CGAAGCTTGATATGAAAAACCATCGTTG-3’ (MVZ 05) and 5’-AAATAGGAARTATCAYTCTGGTTTRAT-3’ (MVZ 16) [14] and of 631 bp of the mitochondrial Cytochrome Oxidase—Subunit I gene was performed with primers 5’-TCAACCAACCACAAAGACATTGGCAC-3’ (FishF1) and 5’-TAGACTTCTGGGTGGCCAAAGAATCA-3’ (FishR1) [15]. Both PCR products were purified with the aid of polyethylene glycol (PEG) and alcohol. Subsequently, sequence reactions were conducted with the BigDye Kit (Applied Biosystems), and the samples were sequenced on the ABI 3500 XL automatic sequencer (Applied Biosystems).

DNA sequences were aligned in ClustalW [16] and manually edited in BioEdit v. 7.2.5 [16]. Phylogenetic analyzes were performed by maximum likelihood (ML). The search for the best nucleotide substitution model was performed using the jModeltest 2.0.2 software [17]. Base saturation tests were performed on DAMBE5 [18] and there was no saturation in the two markers. The ML analysis was performed in RAxML v.8, the most plausible tree was found by 1000 searches and later 1000 bootstrap pseudo-replicas were performed [19]. Percentages of genetic divergence between taxa were verified in the (MEGA) program [20].

### Divergence time analysis and species delimitation

The Automatic Barcode Gap Discovery (ABGD) analysis from the concatenated dataset (Cytb and COI) was performed in the online version (https://bioinfo.mnhn.fr/abi/public/abgd/abgdweb.html), using the following settings: Pmin = 0.001, Pmax = 0.1, Steps = 10, NBins = 20, Relative gap = 0.5, simple distance, the other parameters were not adjusted [21, 22]. The assemble species by automatic partitioning (ASAP) analysis was performed in the online version (https://bioinfo.mnhn.fr/abi/public/asap/) using the distance matrix with the simple distance model (p-distance) and settings standardized [21, 22].

Divergence time analyses were run in BEAST v.1.10 [23]. The uncorrelated relaxed clock [24] was the prior clock type, and the Yule process was the prior model for the tree [25]. We use the fossil of the *Paralonchothrix* dated from 7.0 Ma for the separation between the genera *Mesomys* and *Lonchothrix* [26] as our calibration point. The run was processed with 100,000,000 generations, sampling every 5,000 generations. Trees were summarized in TreeAnnotator v.1.8.4 [23]. A burn-in of 20% was used and the run was considered satisfactory when all Effective Sample Size (ESS) values checked in Tracer v.1.6 [27] were equal to or larger than 200.

### Cytogenetic analysis

Chromosomal preparations were obtained through bone marrow samples [28]. Subsequently, the slides were prepared and submitted to G- [29] and C-banding [30], in addition to FISH (fluorescence *in situ* hybridization) with telomeric and 18S rDNA probes [31]. All techniques followed the established protocols with adaptations.

The slides containing the chromosome preparations used in classical cytogenetic techniques were analyzed under an Olympus BX41 microscope and photographed with a Canon Powershot A95 digital camera. The karyotypes were organized using the Applied Spectral Imaging Karyotyping software, considering the morphology and size of the chromosomes. Previously hybridized slides were captured using Nis-Elements software under the Nikon H550S microscope. Chromosomes are separated into two groups: one-armed (subtelocentric/telocentric/acrocentric) and bi-armed (metacentric/submetacentric).

The chromosomes classification was performed according to Levan et al. [32], which takes into account the quotient between the length of the long chromosomal arm (L) and the short arm (C), as the value of the L/C ratio, with the designations of chromosomes was metacentric, submetacentric, subtelocentric and telocentric due to the position of the centromere, if it occupies the median, submedian, subterminal and terminal positions, respectively.

## Results

### Molecular phylogeny, species delimitation and divergence time tree

**Fig 2:**
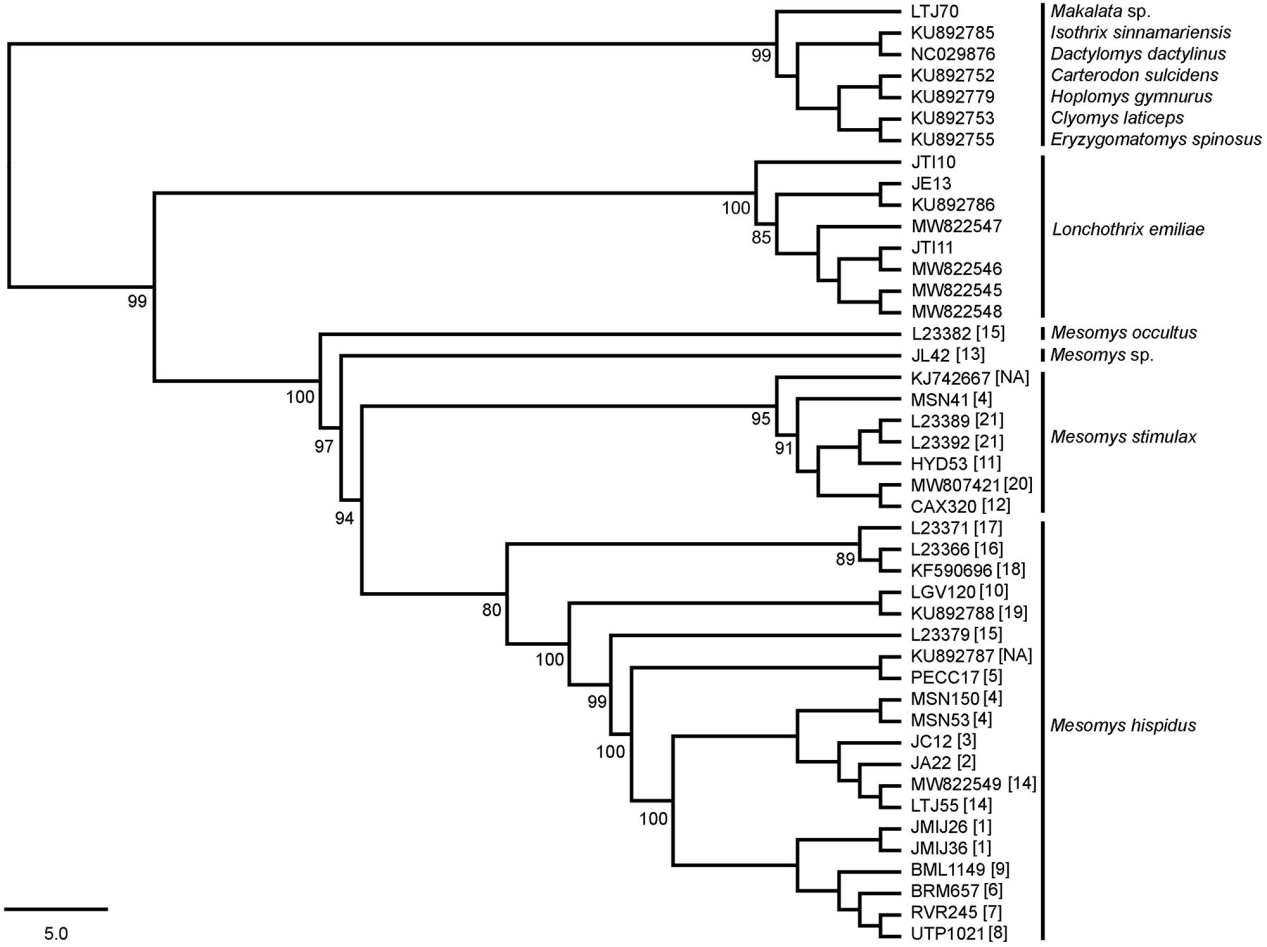
Maximum likelihood tree of the samples from the present study and those available in GenBank for the genus *Mesomys*, based on sequences of concatenated mitochondrial genes (Cytb and COI). The numbers near the nodes are bootstrap values for 1,000 replicates. Bootstrap values less than 80% are not shown. The numbers in square brackets precede the locations are referred to in Fig 1.

Maximum likelihood phylogenetic analysis recovered a topology with high bootstrap values for most nodes. The sister relationship between the genera *Lonchothrix* and *Mesomys* was recovered with 99% bootstrap. The sample from the municipality of Itaituba (Locality 13, Fig 1) did not correspond to any recognized clade of the genus *Mesomys*, and formed a branch with 94% support, suggesting that this is a new species for the genus (Fig 2).

The genetic distance analysis performed with the mitochondrial gene of Cytochrome b (Cytb) showed divergence among *Mesomys* and the other genera ranged from 15% between *M*. *stimulax* and *L*. *emiliae* to 24% between *M*. *occultus* and *I*. *sinnamariensis* (Table 1). Within *Mesomys*, the highest interspecific distance was 14% between *M*. *hispidus* and *M*. *occultus*, and the lowest was 7% for *Mesomys* sp. and *M*. *hispidus*. The latter species showed the highest value of average intraspecific nucleotide divergence (5%). The putative new species of *Mesomys*, herein referred as *Mesomys* sp., differed from the other congeneric species by 7% from *M*. *stimulax* up to 12% from *M*. *occultus* (Table 1).

**Table 1:** P-distance estimates for the Cytochrome b (Cytb) gene among the monophyletic clades recovered in the phylogenetic analysis.

Species	1	2	3	4	5	6	7	8	9	10	11	VI (%)
[1] ***L*. *emiliae***												0.03
[2] ***M*. *hispidus***	0.18											**0.05**
[3] ***M*. *stimulax***	0.15	0.09										**0.04**
[4] ***Mesomys* sp.**	0.16	0.08	**0.07**									--
[5] ***M*. *occultus***	0.16	**0.14**	0.12	0.12								--
[6] ***E*. *spinosus***	0.17	0.21	0.19	0.19	0.21							--
[7] ***D*. *dactylinus***	0.19	0.20	0.20	0.20	0.20	0.18						--
[8] ***C*. *laticeps***	0.21	0.21	0.20	0.20	0.23	0.16	0.19					--
[9] ***H*. *gymnurus***	0.20	0.19	0.20	0.20	0.20	0.20	0.20	0.19				--
[10] ***I*. *sinnamariensis***	0.18	0.23	0.22	0.23	0.24	0.22	0.19	0.22	0.21			--
[11] ***Makalata* sp.**	0.20	0.22	0.21	0.21	0.21	0.19	0.19	0.21	0.22	0.21		--

VI = intraspecific variation. Scores in bold represent the lowest and highest values of interspecific and intraspecific divergence for the different species of *Mesomys* analyzed.

The genetic distance analysis performed using the mitochondrial gene Cytochrome C Oxidase—Subunit I (COI) sequences showed divergence among *Mesomys*, and the other genera ranged from between 12% *Mesomys* sp. and *L*. *emiliae* to 22% between *Mesomys* sp. and *C*. *sulcidens* and also between *E*. *spinosus* and *C*. *sulcidens* (Table 2). Within *Mesomys*, the greatest interspecific distance was 11% between *Mesomys* sp. and *Mesomys hispidus* and also between *M*. *stimulax* and *M*. *hispidus*, and the smallest was 9% for *Mesomys* sp. and *M*. *stimulax*. *Mesomys hispidus* showed the highest value of average intraspecific nucleotide divergence (9%) (Table 2).

**Table 2:** P-distance estimates for the Cytochrome C Oxidase—Subunit I (CO1) gene among the monophyletic clades recovered in the phylogenetic analysis.

Species	1	2	3	4	5	6	7	8	9	10	11	VI (%)
[1] ***L*. *emiliae***												0.04
[2] ***M*. *hispidus***	0.14											**0.09**
[3] ***M*. *stimulax***	0.14	**0.11**										**0.04**
[4] ***Mesomys* sp.**	0.12	**0.11**	**0.09**									--
[5] ***E*. *spinosus***	0.18	0.20	0.20	0.18								--
[6] ***C*. *sulcidens***	0.20	0.21	0.21	0.22	0.22							--
[7] ***D*. *dactylinus***	0.18	0.21	0.19	0.17	0.17	0.20						--
[8] ***C*. *laticeps***	0.19	0.19	0.17	0.19	0.15	0.21	0.18					--
[9] ***H*. *gymnurus***	0.16	0.20	0.20	0.19	0.17	0.21	0.17	0.17				--
[10] ***I*. *sinnamariensis***	0.17	0.19	0.18	0.18	0.17	0.22	0.16	0.18	0.17			--
[11] ***Makalata* sp.**	0.14	0.18	0.19	0.16	0.20	0.20	0.17	0.19	0.18	0.20		--

VI = intraspecific variation. Scores in bold represent the lowest and highest values of interspecific and intraspecific divergence for the different species of *Mesomys* analyzed.

**Fig 3:**
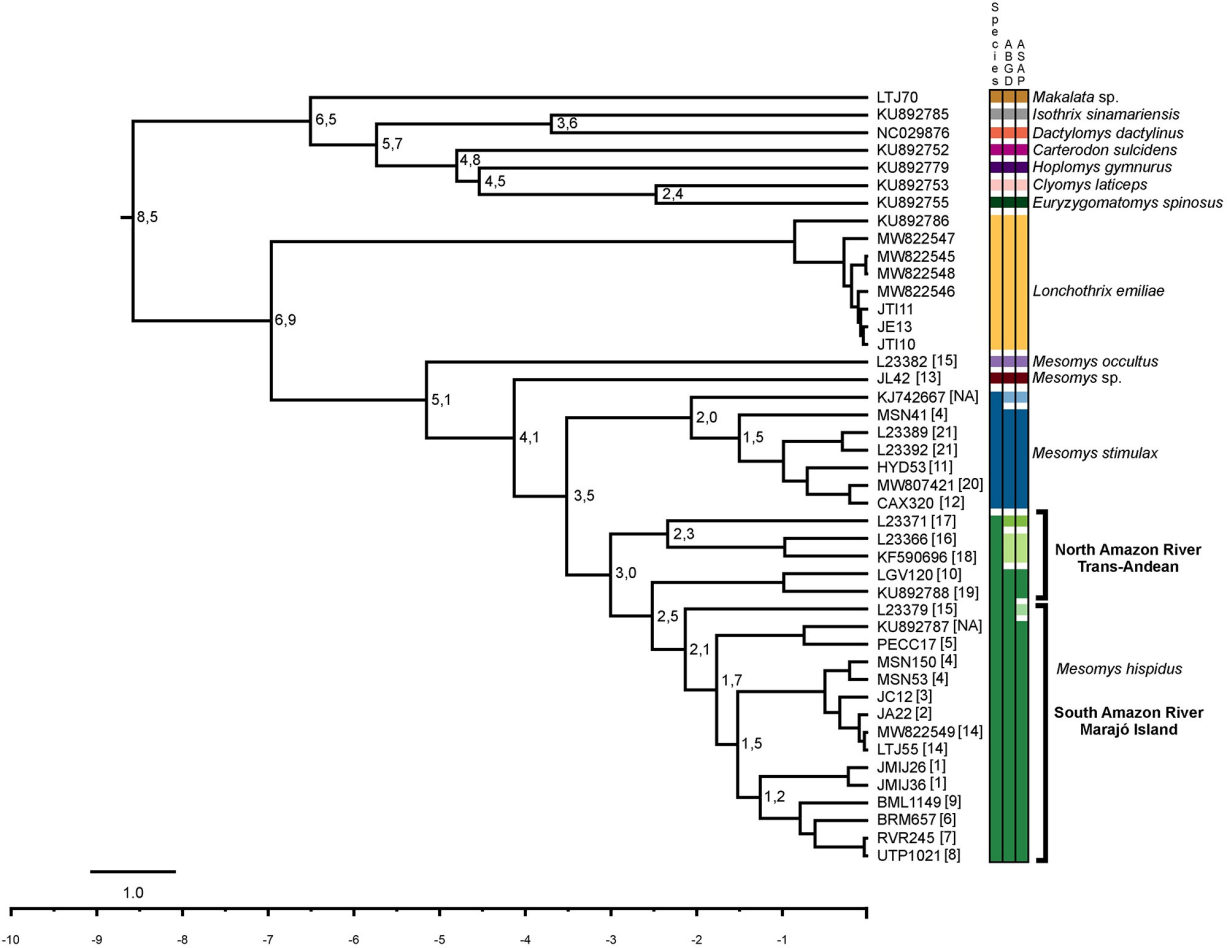
Time tree and species delimitation of the samples from the present study and those available in GenBank for the genus *Mesomys*, based on sequences of concatenated mitochondrial genes (Cytb and COI). Boundaries of 95% confidence intervals: *M*. *occultus* node 5.1 Ma (6.5 Ma– 3.5 Ma), *Mesomys* sp. node 4.1 Ma (5.6 Ma– 2.6 Ma), *M*. *hispidus* + *M*. *stimulax* node 3.5 Ma (4.9 Ma– 2.2 Ma). The numbers in square brackets precede the locations are referred to in Fig 1.

The timetree analysis suggests that the diversification of *M*. *occultus* occurred about 5.1 Ma with error bar (95% HPD: 6.5 Ma– 3.5 Ma) in relation to the other species of the genus. *Mesomys* sp. diverged from *M*. *hispidus* and *M*. *stimulax* about 4.1 Ma (5.6 Ma– 2.6 Ma), and the latter diverged from each other around 3.5 Ma (4.9 Ma– 2.2 Ma) (Fig 3).

The species delimitation performed through the ABGD analysis from the concatenated dataset (Cytb and COI) resulted in the recognition of seven potential species, while the ASAP analysis recovered eight potential species within the genus. *Mesomys occultus* and *Mesomys* sp. were retrieved as valid species in both analyses. *Mesomys stimulax* was recovered as a species complex, composed of two groups in both analyses. Finally, *M*. *hispidus* was also recovered as a species complex, composed of three groups according to ABGD and four groups according to ASAP (Fig 3).

### Classical and molecular cytogenetics

**Fig 4:**
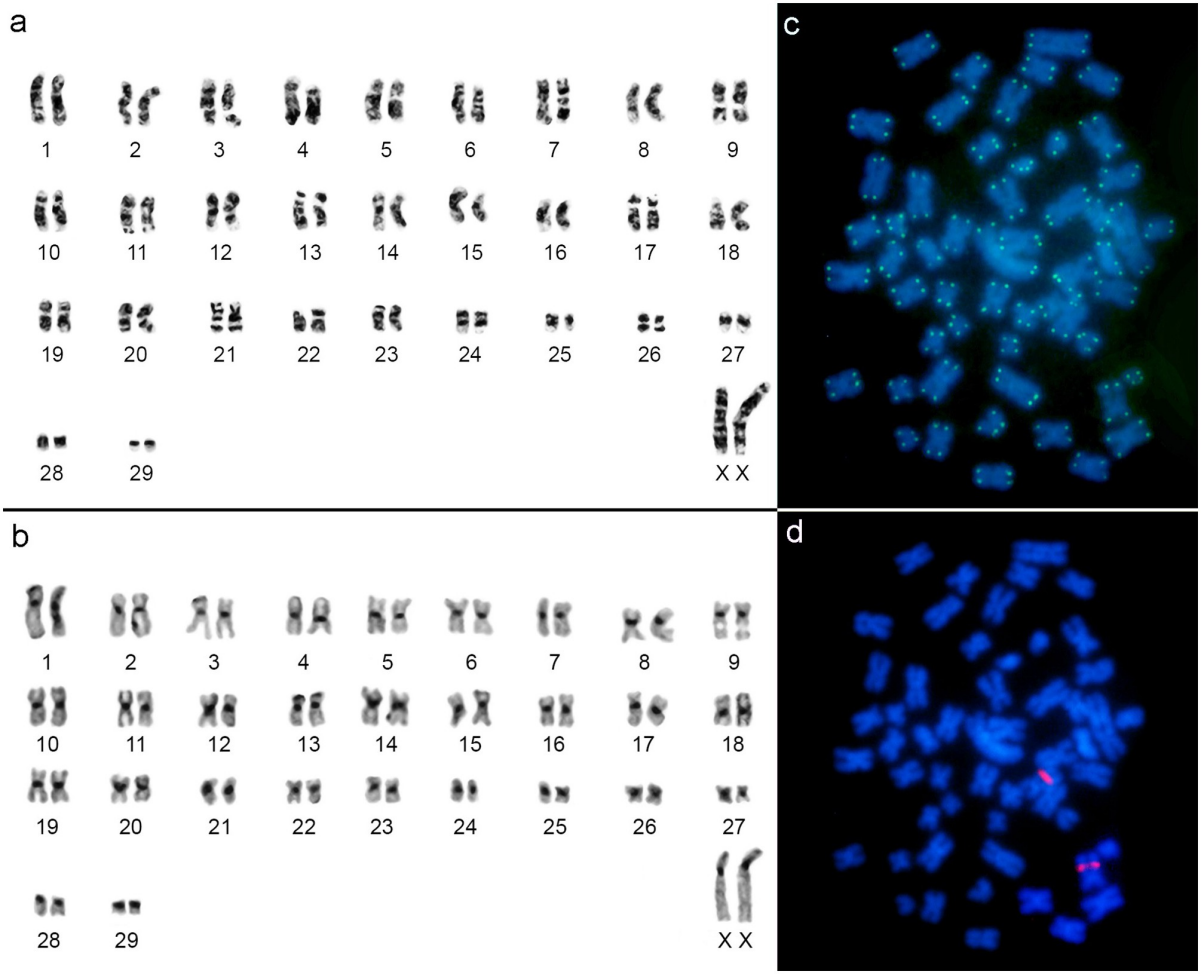
*Mesomys hispidus* (2n = 60/FN = 112). a) G-banding, b) C-banding, c) Telomeric FISH and d) 18S rDNA FISH.

The sample of *Mesomys hispidus* from the municipality of Jacareacanga (Locality 1, Fig 1) presented a karyotype with 2n = 60/FN = 112, with 29 autosomal pairs, ranging from large to small, with pairs 1 to 27 meta/submetacentric, the 28 and 29 acrocentric pairs and the large X chromosome as a submetacentric (Fig 4a). Constitutive heterochromatin (CH) is distributed in the centromeric region of all autosomal and sex chromosomes (Fig 4b). FISH with telomeric probes (Fig 4c) showed only distal signals and those with 18S rDNA probes (Fig 4d) marked in the interstitial region of an autosomal pair.

**Fig 5:**
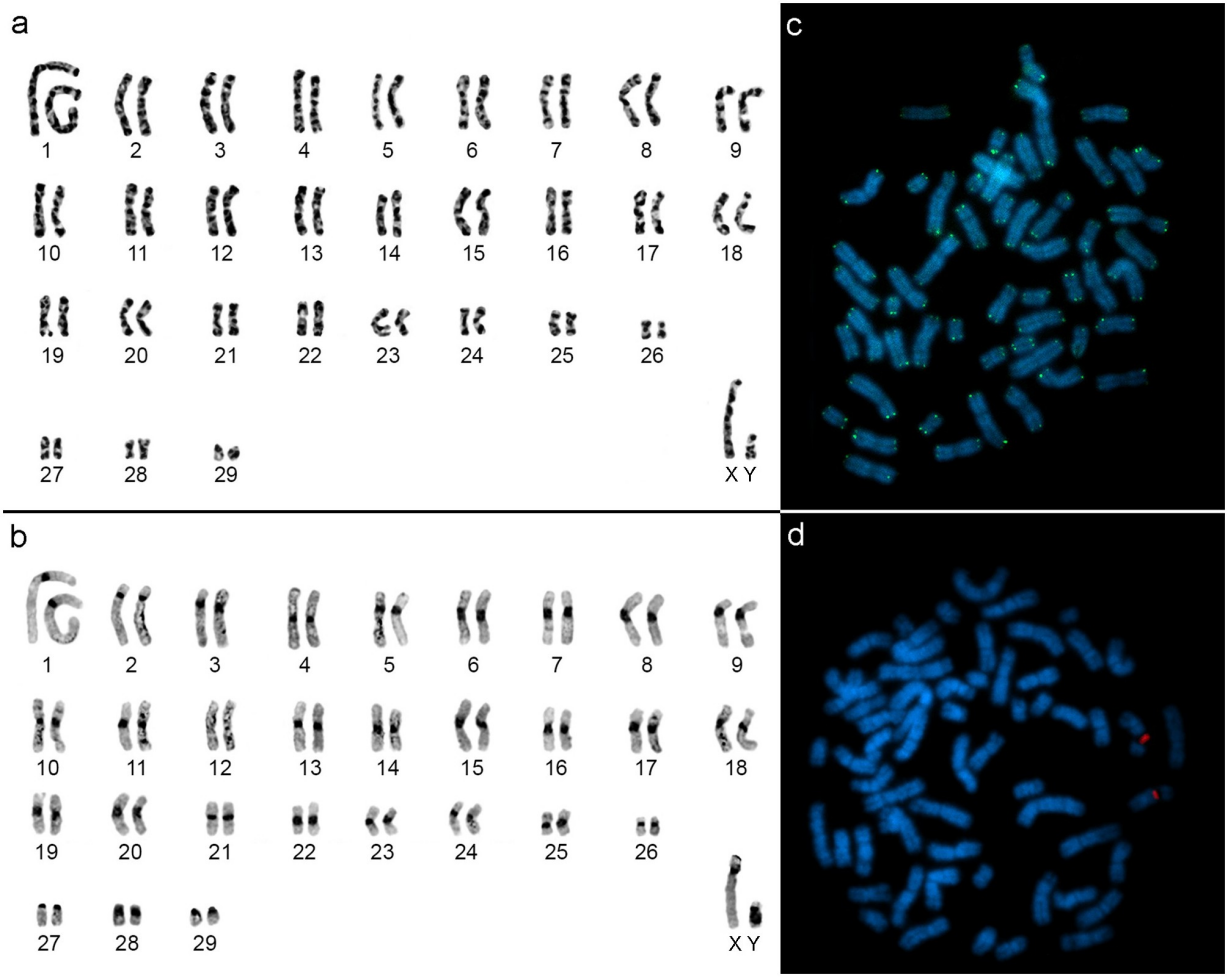
*Mesomys stimulax* (2n = 60/FN = 110). a) G-banding, b) C-banding, c) Telomeric FISH and d) 18S rDNA FISH.

The sample of *Mesomys stimulax* from the municipality of Paragominas (Locality 11, Fig 1) presented the karyotype with 2n = 60/FN = 110, with 29 autosomal pairs, ranging from large to small, with pairs 1 to 26 meta/submetacentric, pairs 27 to 29 acrocentric, a large X chromosome as a submetacentric and the Y as a small submetacentric (Fig 5a). CH is distributed in the centromeric region of all autosomal and sex chromosomes (Fig 5b). FISH with telomeric probes (Fig 5c) showed only distal signals and those with 18S rDNA probes (Fig 5d) marked in the interstitial region of an autosomal pair.

**Fig 6:**
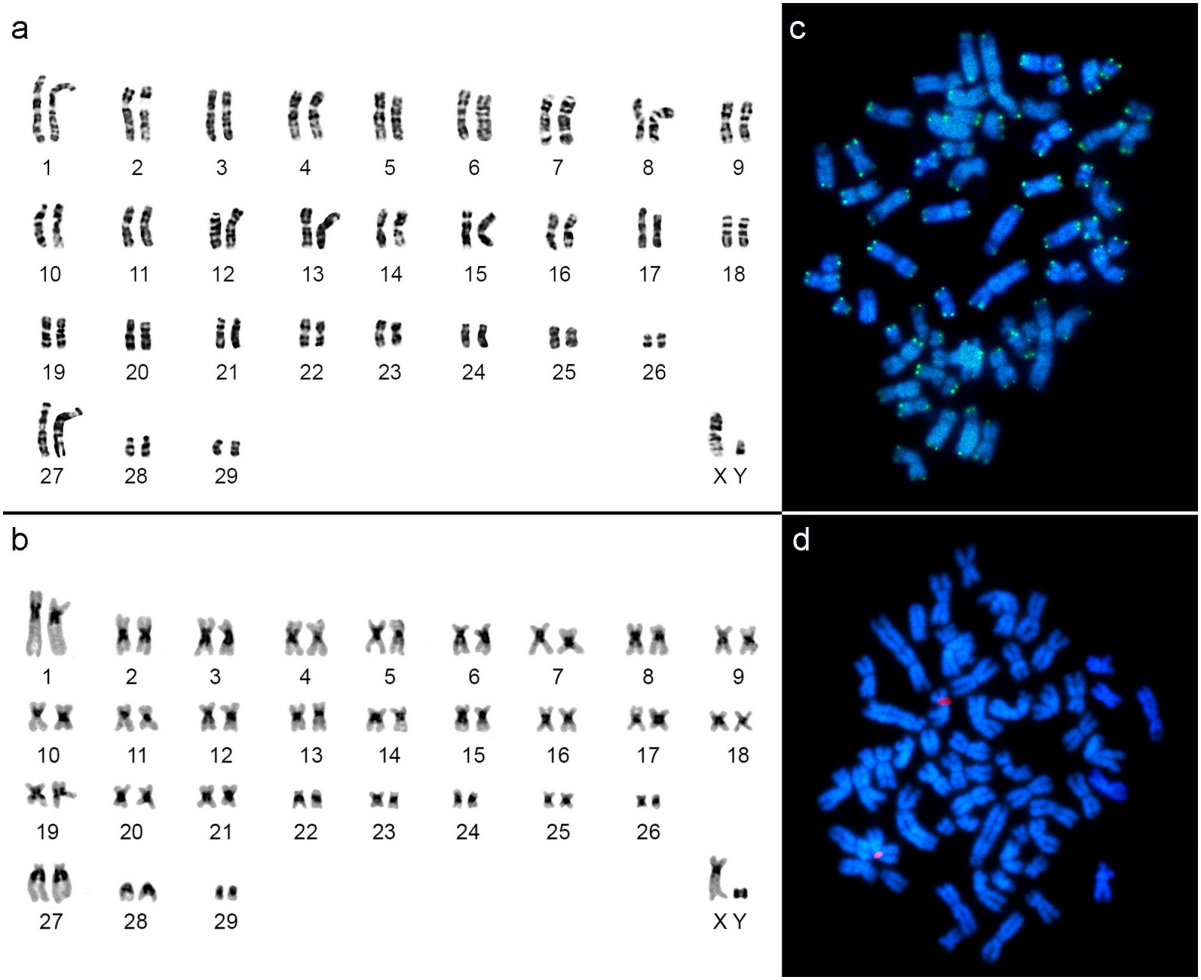
*Mesomys* sp. (2n = 60/FN = 110). a) G-banding, b) C-banding, c) Telomeric FISH and d) 18S rDNA FISH.

The sample of *Mesomys* sp. from the municipality of Itaituba (Locality 13, Fig 1), presented the karyotype with 2n = 60/FN = 110, with 29 autosomal pairs, ranging from large to small, with pairs 1 to 26 meta/submetacentric, pair 27 subtelocentric, pairs 28 and 29 acrocentric, a large X chromosome as a submetacentric and the Y as a small metacentric (Fig 6a). CH is distributed in the proximal region of all autosomal and sex chromosomes (Fig 6b). FISH with telomeric probes (Fig 6c) showed only distal signals and those with 18S rDNA probes (Fig 6d) marked in the interstitial region of an autosomal pair.

## Discussion

### Phylogenetic analysis and species delimitation

Maximum likelihood (ML) analysis using the concatenated Cytb + COI markers showed the monophyly of the genus *Mesomys* well supported, corroborating the literature data [7, 8]. Our samples of *M*. *hispidus* and *M*. *stimulax* were correctly associated with the sequences available in GenBank for these species, confirming the taxonomic identification from DNA barcoding [33–35].

A lineage unknown to the genus *Mesomys* for the sample from the Tapajós endemic area, in the municipality of Itaituba-PA, proves to be a sister lineage of the *M*. *hispidus* + *M*. *stimulax* grouping with 97% and it was recovered diverging in a branch with 94% support. The interspecific divergence based on the mitochondrial Cytb gene between the *Mesomys* species included in this study and the new lineage detected ranged between 7% and 12%, while the divergence values recorded for the mitochondrial COI gene ranged from 9 to 11% (Tables 1 and 2, respectively). Reinforcing the findings of the ML analysis, the species delimiters ABGD and ASAP (Fig 3) support the hypothesis of *Mesomys* sp. (JL42) as a distinct lineage.

According to the average intraspecific variation for the analyzed species, *M*. *stimulax* was the one that presented the lowest rate, with 4% in both genes (Cytb and COI) for seven samples from five localities. The species *M*. *hispidus* showed the highest intraspecific nucleotide divergence with 5% for Cytb and 9% for COI for twenty samples from sixteen localities. The high intraspecific divergence found in *M*. *hispidus* corroborates other studies, where clades with 7.2% nucleotide divergence were described, three of which are widely distributed and three are geographically isolated in the Amazon region [9–11]. Values above 5% of nucleotide divergence for mitochondrial genes in rodents are indicative of possible cryptic species [36, 37].

Furthermore, we observed the formation of geographically structured clades within *Mesomys hispidus* congruent with species delimiters. The first subclade to diverge about 3 Ma from the other branches in *M*. *hispidus* points to two potential species according to the ABGD and ASAP delimiters; the first corresponding to the sample from the Guyana shield (Locality 17, Fig 1), and the second corresponding to samples from the trans-Andean region (Localities 16 and 18, Fig 1), diverging from each other at approximately 2.3 Ma. Together with the samples from locations 10 and 19 (Fig 1) these two possible species appear to be older groups related to the north of the Amazon River. Meanwhile, another large subclade associated with samples south of the Amazon River appears to have its most recent diversification splitting from samples to the north at approximately 2.5 Ma (Fig 3). Despite the low support of nodes (80% bootstrap), the existence of these groups evidenced by species delimiters reinforces the idea of species complexes in *M*. *hispidus*, as already postulated [9–11, 38].

### Geographical patterns, divergence time and speciation hypotheses

The genus *Mesomys* is widely distributed in the Amazon and in the eight areas of endemism. *Mesomys leniceps* occurs in the Napo area of endemism; *M*. *occultus* occurs in the areas of endemism Napo and Inambari; *M*. *hispidus* is distributed in six areas of endemism, with the exception of Belém and Xingu areas; and *M*. *stimulax* is distributed in the Xingu, Tapajós, Rondônia, Belém and Guianas areas of endemism [6, 11, 12].

**Fig 7:**
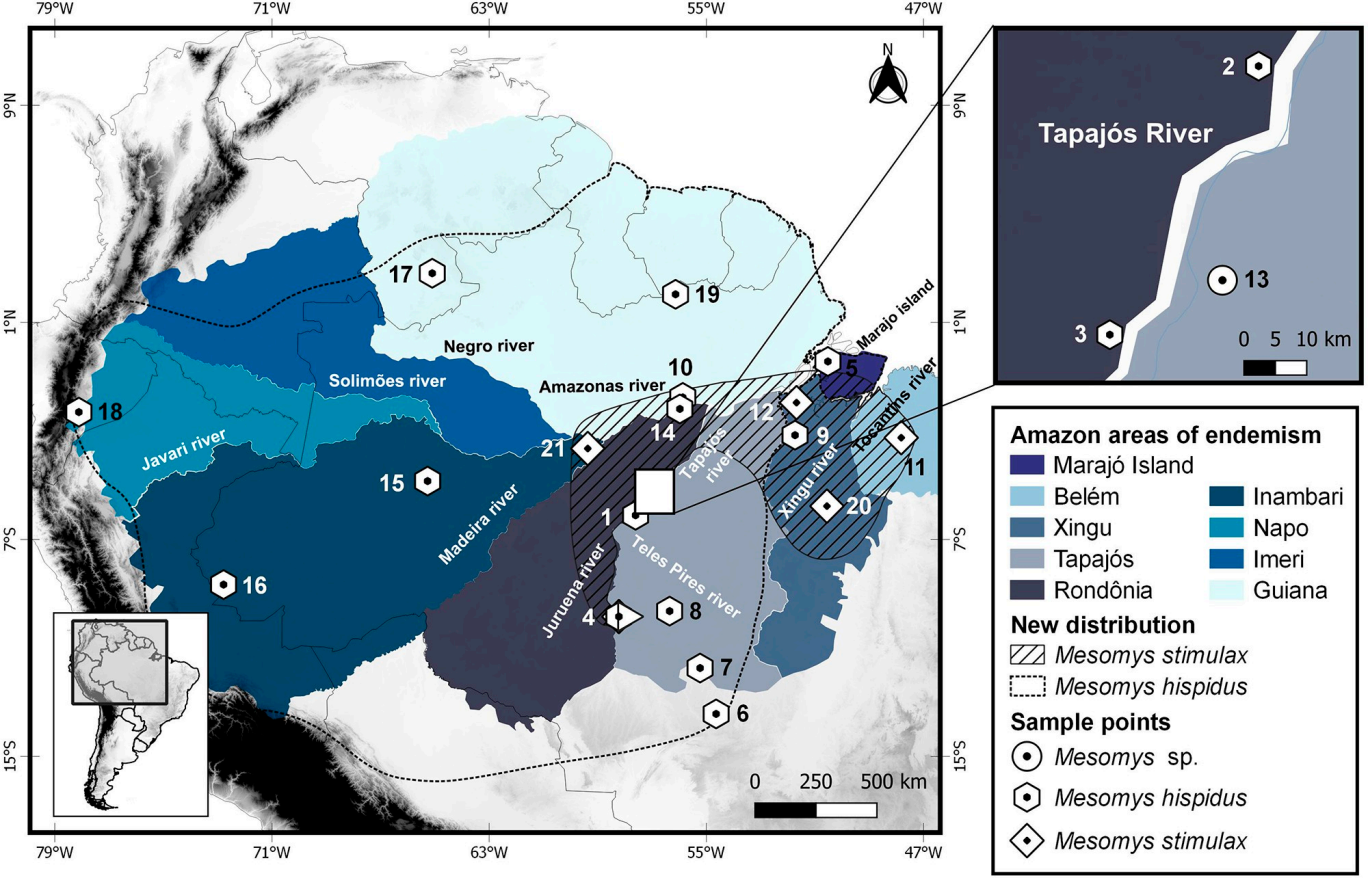
Proposal for expanding the geographic distribution for the species *M*. *hispidus* and *M*. *stimulax*. Circle: *Mesomys* sp., hexagon: *M*. *hispidus*, diamond: *M*. *stimulax* and square: *M*. *occultus*. This map was made using the free software Quantum-Gis version 2.10.1. The databases were obtained from DIVA-GIS. The files provided by DIVA-GIS are free of charge.

Regarding the distribution and sympatry between the species *M*. *hispidus* and *M*. *stimulax*, studies describe *M*. *hispidus* with the eastern limit of occurrence on the left bank of the Cristalino river, affluent of the right bank of the Teles Pires river, and *M*. *stimulax* occurring in part of central and eastern Amazonia, from the west bank of the lower Tapajós River to the east bank of the Tocantins River [6]. However, our analysis extends the distribution of *M*. *hispidus* to the right bank of the middle Tapajós river, Pará state, on both banks of the middle and upper Teles Pires river, Mato Grosso state, and Marajó island, Pará state, in the areas of endemism Tapajós and Marajó Island, respectively. Regarding *M*. *stimulax*, our analysis extends the occurrence of this species to the west of the Amazon, on the west bank of the lower Madeira river, state of Amazonas, and the left bank of the river Juruena, state of Mato Grosso, in the areas of endemism Inambari and Rondônia, respectively. Thus, we propose to expand the distribution of these species to these locations not recognized so far (Fig 7).

Recent studies have described new species for the Tapajós region, corroborating its characterization as an endemic area, increasing the number of endemic species of different taxonomic groups for the region, including small non-flying mammals [39–42]. Some of these studies proposed the hypothesis that the Amazon rivers act as a geographic barrier, being the main factor that isolated the populations, influencing the differentiation of the species that occur in this area of endemism [41–44]. On the other hand, the hypothesis of rivers as a barrier is questioned, suggesting that the modifications depend on the characteristics of each river and the studied species [45, 46].

Analysis of mitochondrial sequences of *Mesomys hispidus* collected along the Juruá river tested the hypothesis of the river as a barrier [38]. In this study, clades grouping samples from both banks of the river were described and the gene flow data were uncertain to distinguish the intensity with which they occurred on each bank. Thus, they concluded that the Juruá River was not acting as a barrier for *M*. *hispidus*.

With regard to the molecular dating of arboreal Echimyidae, the time of diversification is estimated for the Miocene, between 17.1 and 15.3 Ma, being related to vicariant events, such as the emergence of river drainage and the formation of flooded forests, which acted on the ecomorphological modifications of these taxa, modifying the way of life for the tree extract [6–8, 47–49]. Our divergence time data show that *Mesomys* diversification started around 5.1–3.5 Ma during the transition from the Pliocene to the Pleistocene period, before the formation of the Tapajós River, which occurred around 1.3–0.8 Ma, and this physical barrier is not the determining factor for radiation in the genus [44].

A phylogenetic and biogeographical analysis of rodent radiation in the transition from Amazonian and Andean centers of endemism suggested that diversification between species of the genus occurred between 7 Ma– 2.5 Ma, while more recent populations are observed ranging from 4 Ma– 1 Ma, possibly influenced by Plio-Pleistocene climate changes, causing them to become isolated [50]. In this sense, our data are congruent with those described [50], where the analyzed species diversified between 6.5 Ma– 2.2 Ma and the populations varied between 3.2 Ma– 1 Ma, which allows us to indicate the influence of effects that occurred in the Plio-Pleistocene period.

In view of the difficulties of taxonomic identification and the molecular issues associated with the genus *Mesomys*, multidisciplinary approaches play a role in elucidating the diversity and evolution of this understudied genus [51, 52]. Additionally, it is worth noting that different rodent groups exhibit cryptic species, and the use of these tools becomes crucial in identifying potential new species [53].

### Cytogenetic analysis

The data of the present work show a new cytotype for *M*. *hispidus*, from the municipality of Jacareacanga-PA (Locality 1, Fig 1), having 2n = 60/FN = 112. This sample has two acrocentric pairs (28 and 29), which differs from the karyotypes described in the literature for *M*. *hispidus* from Juruti-PA (Locality 14, Fig 1) [11] and *M*. *hispidus* from the upper Juruá river [9] that present all chromosomal pairs with bi-armed morphology (2n = 60/FNa = 116). The conservation of 2n = 60, with variation in NF = 112 and 116 may be due to chromosomal rearrangements like pericentric inversion, being one of the chromosomal variations common in rodents [54–56].

*Mesomys stimulax* with 2n = 60/FNa = 110 from the municipality of Paragominas-PA (Locality 11, Fig 1), corroborates the diploid number (2n = 60) and number of autosomal arms (FNa = 110) previously described [12] (Locality 20, Fig 1). However, it presents CH variation, which is restricted in the centromeric region, while *M*. *stimulax* presents CH blocks in the centromeric region, proximal region, having some pairs almost all heterochromatic [12].

The sample of *Mesomys* sp. from the municipality of Itaituba-PA (Locality 13, Fig 1) presents 2n = 60 and FN = 110 similar to the karyotype described for *M*. *stimulax* from Paragominas-PA (Locality 11, Fig 1) and Marabá-PA (Locality 20, Fig 1) [12]. However, it differs in chromosomal morphology, presenting one large subtelocentric chromosome pair, while the other populations have three small acrocentric pairs. Furthermore, it differs in the karyotypic formula, with *M*. *stimulax* exhibiting 21 metacentric + 5 submetacentric + 3 acrocentric (20m + 5 sm + 3a), while *Mesomys* sp. shows 18 metacentric + 8 submetacentric + 1 subtelocentric + 2 acrocentric (18m + 8sm + 1st + 2a). The comparative analysis by C-banding also revealed a pattern of proximal distribution of CH in the autosomes and sex chromosomes for *Mesomys* sp., similar to that described for *M*. *stimulax* from Marabá-PA (Locality 20, Fig 1) in some pairs, however different from *M*. *stimulax* from the population of Paragominas-PA (Locality 11, Fig 1) in which the CH pattern is restricted to the centromeric region [11, 12].

The data here described for FISH with telomeric probe and 18S rDNA corroborated what was described in the literature for populations of *M*. *hispidus* from Juruti-PA (Locality 14, Fig 1) and *M*. *stimulax* from Marabá-PA (Locality 20, Fig 1), showing distal telomeric signals and 18S rDNA signal in a chromosomal pair [11, 12].

Thus, the phylogenetic and karyotypic differences found in *Mesomys* sp. (2n = 60/NF = 110) may be related to the Amazon refuge hypothesis due to multiple ecological and biogeographical events that occurred during the Plio-Pleistocene periods (11.7 Ma– 2.5 Ma) acting on the dispersion and isolation of populations. A study carried out [61] pointed to hypotheses of sympatric and allopatric speciation in rodents of the genus *Proechimys* in the endemic areas of Rondônia and Tapajós, suggesting that chromosomal rearrangements that occurred within subpopulations established in allopatry could serve to reinforce the blockage of gene flow in conditions of secondary contact. In this case, considering that *Mesomys* sp. also occurs in the Tapajós area of endemism in sympatry with *Mesomys hispidus*, the chromosomal differences observed between these species may be associated with vicariant effects that allowed the occurrence of independent chromosomal rearrangements in these sister species [8, 57–61].

## Conclusions

We described a new cytotype for *M*. *hispidus* from Jacareacanga with karyotypic form 2n = 60/NF = 112, unpublished for the genus. Our molecular and phylogenetic data revealed a new lineage for the genus *Mesomys* based on a sample from the municipality of Itaituba-PA, within the Tapajós area of endemism. This new lineage is probably in sympatry with the species *M*. *hispidus* and *M*. *stimulax*, suggesting that it is a species not yet described for the genus, here called *Mesomys* sp. nov. Furthermore, *M*. *hispidus* showed a high degree of intraspecific divergence, suggesting that it may represent a species complex. Based on our data, we propose expanding the geographic distribution of *M*. *hispidus* to the right bank of the middle Tapajós River, state of Pará, to both margins of the middle and upper Teles Pires River, state of Mato Grosso, and to the Marajó island, state of Pará. Additionally, we propose expanding *M*. *stimulax* geographic distribution to the western Amazonia, particularly on the lower Madeira River, state of Amazonas, and on the banks of the Juruena River, state of Mato Grosso, in the Inambari and Rondônia areas of endemism, respectively.

## Supporting information

S1 Table List of specimens of the genus *Mesomys* included in the molecular analysis of Cytochrome b (Cytb) and Cytochrome C Oxidase—Subunidade I (CO1) whose sequences were employed in this study. For each sample, the GenBank number/Voucher, locality and reference are provided.
(DOCX)

S2 Table Information on 2n (diploid number), FN (fundamental number) and the locality of the Mesomys whose karyotypes were employed in this study. The number that precedes the locations are referred in Fig 1.
(DOCX)
