# Supplementary material for: Genetic diversity analysis in the Brazilian Amazon reveals a new evolutionary lineage and new karyotype for the genus Mesomys (Rodentia, Echimyidae, Eumysopinae)
Source: PLoS One. 2023 Oct 4;18(10):e0291797. doi: 10.1371/journal.pone.0291797 (PMC10550160; doi:10.1371/journal.pone.0291797)
Supplement: S1 Table — For each sample, the GenBank number/Voucher, locality and reference are provided. (DOCX) [file pone.0291797.s001.docx]

**Supplementary Table 1.** List of specimens of the genus *Mesomys* included in the molecular analysis of Cytochrome b (Cytb) and Cytochrome C Oxidase - Subunidade I (CO1) whose sequences were employed in this study. For each sample, the GenBank number/Voucher, locality and reference are provided.

| **Species** | **Collection number** | **Voucher number** | **GenBank** | **Locality** | **Reference** |
| --- | --- | --- | --- | --- | --- |
| *Mesomys hispidus* |  | JMIJ26 |  | [1] Jacareacanga, Pará, Brazil | Present work |
|  | UFPAM1158 | JMIJ36 |  | [1] Jacareacanga, Pará, Brazil | Present work |
|  | UFPAM1551 | JA22 |  | [2] Itaituba, Pará, Brazil | Present work |
|  | UFPAM1159 | JC12 |  | [3] Itaituba, Pará, Brazil | Present work |
|  |  | MSN53 |  | [4] Cotriguaçu, Mato Grosso, Brazil | Present work |
|  |  | MSN150 |  | [4] Cotriguaçu, Mato Grosso, Brazil | Present work |
|  |  | PECC17 |  | [5] Afuá, Ilha do Marajó, Pará, Brazil | Present work |
|  |  | BRM657 |  | [6] Nova Ubiratã, Mato Grosso, Brazil | Present work |
|  |  | RVR245 |  | [7] Sinop, Mato Grosso, Brazil | Present work |
|  |  | UTP1021 |  | [8] Paranaíta, Mato Grosso, Brazil | Present work |
|  |  | BML1149 |  | [9] Vitória do Xingu, Pará, Brazil | Present work |
|  |  | LGV120 |  | [10] Oriximiná, Pará, Brazil | Present work |
|  |  | LTJ55 |  | [14] Juruti, Pará, Brazil | Present work |
|  |  | LTJ65 | MW822549 | [14] Juruti, Pará, Brazil | Dias de Oliveira et al. (2019) |
|  |  | MNFS188 | L23379 | [15] Amazonas, Brazil | Da Silva and Patton (1993) |
|  | MPEG28606 | MNFS1230 | L23366 | [16] Acre, Brazil | Da Silva and Patton (1993) |
|  |  | ALG14162 | L23371 | [17] Amazonas, Brazil | Da Silva and Patton (1993) |
|  | MEPN12212 |  | KF590696 | [18] Morona Santiago, Ecuador | Upham et al. (2013) |
|  | RMNH.MM.21728 |  | KU892788 | [19] Sipaliwine, Suriname | Fabre et al. (2016) |
|  | T6523 |  | KU892787 |  | Fabre et al. (2016) |
| *Mesomys stimulax* |  | HYD53 |  | [11] Paragominas, Pará, Brazil | Present work |
|  |  | MSN41 |  | [4] Cotriguaçu, Mato Grosso, Brazil | Present work |
|  |  | CAX320 |  | [12] Caxiuanã, Pará, Brazil | Present work |
|  | MPEG42030 | PSA188 | MW807421 | [20] Marabá, Pará, Brazil | Malcher et al. (2021) |
|  | USNM549807 | MDC550 | L23389 | [21] Altazes, Amazonas, Brazil | Da Silva and Patton (1993) |
|  | USNM549808 | LHM572 | L23392 | [21] Altazes, Amazonas, Brazil | Da Silva and Patton (1993) |
|  |  | UFROM379 | KJ742667 |  | Upham and Patterson (2014) |
| *Mesomys occultus* |  | MNFS201 | L23382 | [15] Coari, Amazonas, Brazil | Da Silva and Patton (1993) |
| *Mesomys* sp. |  | JL42 |  | [13] Itaituba, Pará, Brazil | Present work |
