# Supplementary material for: Genetic diversity analysis in the Brazilian Amazon reveals a new evolutionary lineage and new karyotype for the genus Mesomys (Rodentia, Echimyidae, Eumysopinae)
Source: PLoS One. 2023 Oct 4;18(10):e0291797. doi: 10.1371/journal.pone.0291797 (PMC10550160; doi:10.1371/journal.pone.0291797)
Supplement: S2 Table — The number that precedes the locations are referred in Fig 1. (DOCX) [file pone.0291797.s002.docx]

**Supplementary Table 2.** Information on 2n (diploid number), FN (fundamental number) and the locality of the *Mesomys* whose karyotypes were employed in this study. The number that precedes the locations is referred in Fig 1.

| **Species** | **2n** | **FN** | **Locality** | **Reference** |
| --- | --- | --- | --- | --- |
| *Mesomys hispidus* | 60 | 112 | [1] Jacareacanga, Pará, Brazil | Present work |
| *Mesomys hispidus* | 60 | 116 | Amazonas, Brazil | Patton et al. (2000) |
| *Mesomys hispidus* | 60 | 116 | [14] Parintins, Amazonas, Brazil | Dias de Oliveira et al. (2019) |
| *Mesomys stimulax* | 60 | 116 | Pará, Brazil | Patton et al. (2000) |
| *Mesomys stimulax* | 60 | 110 | [11] Paragominas, Pará, Brazil | Present work |
| *Mesomys stimulax* | 60 | 110 | [20] Parauapebas, Pará, Brazil | Malcher et al. (2021) |
| *Mesomys* sp. | 60 | 110 | [13] Itaituba, Pará, Brazil | Present work |
| *Mesomys occultus* | 42 | 54 | Upper Urucu river, Amazonas, Brazil | Patton et al. (2000) |

**References**

Dias de Oliveira L., Oliveira da Silva W., Rodrigues da Costa MJ, Sampaio I, Pieczarka JC, Nagamachi CY (2019). First cytogenetic information for *Lonchothrix emiliae* and taxonomic implications for the genus taxa *Lonchothrix* + *Mesomys* (Rodentia, Echimyidae, Eumysopinae). PLoS ONE, 14(4): e0215239. https://doi.org/10.1371/journal.pone.0215239

Fabre PH, Upham NS, Emmons LH, Justy F, Leite YL, Loss AC, Orlando L, Tilak MK, Patterson BD, Douzery EJ (2016). Mitogenomic phylogeny, diversification, and biogeography of South American spiny rats. Molecular Biology and Evolution, 34 (3): 613-633.

Leite YL, Patton JL (2002). Evolution of South American spiny rats (Rodentia, Echimyidae): the star-phylogeny hypothesis revisited. Molecular Phylogenetics and Evolution, 25 (3): 455-464.

Malcher SM, Pieczarka JC, Pereira AL, Amaral PJS, Rossi RV, Saldanha et al. New karyotype for *Mesomys* *stimulax* (Rodentia, Echimyidae) from the Brazilian Amazon: A case for species complex? Ecology and Evolution. 2021, v. 11, n. 12, p. 7125-7131.

Patton JL, Silva MNF, Malcolm JR. Mammals of the Rio Juruá and the Evolutionary and ecological diversification of Amazonia. Bulletin of the American Museum of Natural History. 2000, vol. 97, no. 25.

Silva MNF, Patton JL (1993). Amazonian phylogeography: mtDNA sequence variation in arboreal. Molecular Phylogenetics and Evolution, 2: 243-255.

Upham NS, Patterson BD (2014). Phylogeny and evolution of caviomorph rodents (Rodentia, Hystricomorpha): a complete timetree for living genera. Pp. 63-120 in Vassallo, A.I., Antenucci, D. (editors). Biology of caviomorph rodents: diversity and evolution. SAREM Series A, Buenos Aires.
